# Mitogen-activated protein kinase eight polymorphisms are associated with immune responsiveness to HBV vaccinations in infants of HBsAg(+)/HBeAg(−) mothers

**DOI:** 10.1186/s12879-018-3166-x

**Published:** 2018-06-14

**Authors:** Meng Zhuo Cao, Yan Hua Wu, Si Min Wen, Yu Chen Pan, Chong Wang, Fei Kong, Chuan Wang, Jun Qi Niu, Jie Li, Jing Jiang

**Affiliations:** 1 Division of Clinical Research, The First Hospital of Jilin University, Changchun, 130021 Jilin China; 2 Department of Microbiology and Infectious Disease Center, School of Basic Medical Sciences, Peking University Health Science Center, Beijing, 100020 China; 3 Division of Education, Beijing Jishuitan Hospital, Beijing, 100020 China; 4 Department of Hepatology, The First Hospital of Jilin University, Changchun, 130021 Jilin China; 5 Maternal and Child Health Center of Chaoyang District, Beijing, 100020 China

**Keywords:** Hepatitis B vaccination, MAPK8, TNF, Mother-to-infant transmission, Low response

## Abstract

**Background:**

Infants born to hepatitis B surface antigen (HBsAg) positive mothers are at a higher risk for Hepatitis B virus (HBV) infection. Host genetic background plays an important role in determining the strength of immune response to vaccination. We conducted this study to investigate the association between Tumor necrosis factor (TNF) and Mitogen-activated protein kinase eight (MAPK8) polymorphisms and low response to hepatitis B vaccines.

**Methods:**

A total of 753 infants of HBsAg positive and hepatitis Be antigen (HBeAg) negative mothers from the prevention of mother-to-infant transmission of HBV cohort were included. Five tag single nucleotide polymorphism (SNPs) (rs1799964, rs1800629, rs3093671, rs769177 and rs769178) in *TNF* and two tag SNPs (rs17780725 and rs3827680) in *MAPK8* were genotyped using the MassARRAY platform.

**Results:**

A higher percentage of breastfeeding (*P* = 0.013) and a higher level of Ab titers were observed in high responders (*P* < 0.001). The *MAPK8* rs17780725 AA genotype increased the risk of low response to hepatitis B vaccines (OR = 3.176, 95% CI: 1.137–8.869). Additionally, subjects with the AA genotype may have a lower Ab titer than subjects with GA or GG genotypes (*P* = 0.051). Compared to infants who were breastfed, infants who were not breastfed had an increased risk of low response to hepatitis B vaccine (OR = 2.901, 95% CI:1.306–6.441).

**Conclusions:**

*MAPK8* polymorphisms are associated with immune response to HBV vaccinations in infants of HBsAg(+)/HBeAg(−) mothers.

**Electronic supplementary material:**

The online version of this article (10.1186/s12879-018-3166-x) contains supplementary material, which is available to authorized users.

## Background

Hepatitis B virus (HBV) infection is a global health problem with 780,000 related deaths annually [1]. Mother-to-child transmission (MTCT) of HBV remains a major cause of chronic HBV infection. The most effective way to avoid MTCT is by carrying out a standardized and sufficient hepatitis B immunization program in newborns [2, 3]. In China, from 1992 to 2006, the prevalence of hepatitis B surface antigen (HBsAg) has been reduced by 90% among children under 5 years old, as a result of an HBV immunoprophylaxis program [4]. However, approximately 6–20% of infants born to HBsAg(+) mothers exhibited a non- or low- response to the HBV vaccine, which led to increased risk of horizontal transmission of HBV infection [5–8]. Vaccine type, low birth weight and high maternal viral load have been identified as the most important risk factors for low immune response to HBV vaccine [9–11]. In addition, host genetic background also plays an important role in determining the strength of immune response to vaccination.

Several genome-wide association studies (GWAS) have identified variants in human leukocyte antigen (HLA) region associated with HBV infection and with responsiveness to HBV vaccination due to their important role of immune regulation [12–14]. However, as the genetic variation in the HLA region can only explain the 50% of the susceptibility to low vaccination response, other genetic variants outside this complex also need to be investigated [15]. Tumor necrosis factor (TNF) is a well-characterized pro-inflammatory cytokine that plays a central role through direct immunomodulatory actions. It is also known as an activator of the MAPK and NF-κB signaling pathways, leading to the up-regulation of the immune response [16, 17]. In addition, single nucleotide polymorphism (SNPs) in *TNF* and *MAPK8* have been reported to correlate with peak anti-HBs level in Whites [18, 19]. However, the correlations between these SNPs and immune response to vaccination in a Chinese population remain uncertain.

To better explore the effects of *TNF* and *MAPK* polymorphisms on the response to hepatitis B vaccination, we conducted a case-control study in our MTCT cohort. All of the participants were infants of HBsAg(+)/HBeAg(−) mothers. These infants had an increased risk of HBV infection due to vertical and horizontal transmission. We selected tag SNPs of *TNF* and *MAPK* and genotyped these SNPs using the MassARRAY platform. We then investigated the association between our tag SNPs and low response to hepatitis B vaccination, as well as to Antibody (Ab) titers.

## Methods

### Subjects

**Fig. 1:**
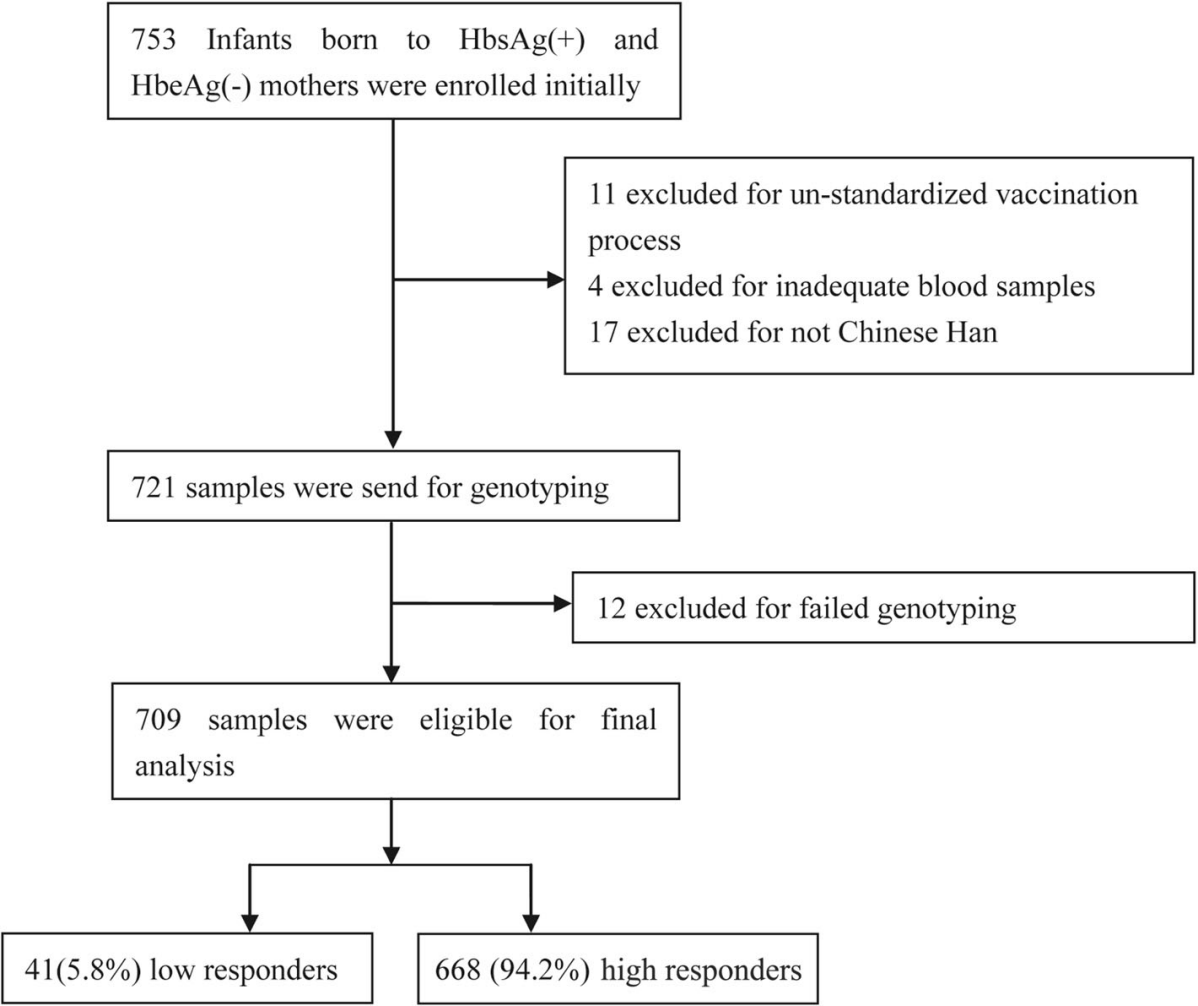
Flow chart of the study

A total of 753 Infants born to HbsAg(+) and HbeAg(−) mothers were enrolled initially from a cohort of mother-to-child transmission of HBV at the First Hospital of Jilin University from July 2012 to July 2015 [1]. We excluded infants with a non-standardized vaccination process, inadequate blood samples, racial background other than Han Chinese and failed genotyping. A total of 709 samples were eligible for our final analysis (Fig. 1). All participants gave written informed consent. The signed consent was obtained from a parent or guardian on behalf of their infants. Our study was approved by the Medical Ethics Committee of the First Hospital of Jilin University (approval number 2012–098).

### Vaccination and laboratory examinations

Infants were injected with 100 IU HBIG (Hualan Biological Engineering, Xinxiang, China) and 10 μg recombinant vaccine (Hansenula polymorpha yeast-derived recombinant hepatitis B vaccine; Dalian Hissen BioPharm, Dalian, China) within 2 h after birth, followed by administration of the same dose at 1 month and 6 months of age. Blood samples from infants were collected at the 7-month follow-up visit (1 month after the last HBV vaccination). Serum samples from infants were sent to the same central laboratory for quantification of HBV markers by chemiluminescent microparticle immunoassay (CMIA) with Abbott ARCHITECT i2000SR (Abbott Laboratories, North Chicago, IL). Other covariates were collected through phone calls, including infant gender, birth weight, gestational age, delivery mode and feeding pattern.

### SNP selection and genotyping

Five tag SNPs (rs1799964, rs1800629, rs3093671, rs769177 and rs769178) in *TNF* and two tag SNPs (rs17780725 and rs3827680) in *MAPK8* were selected from two online databases, GVS (http://gvs.gs.washington.edu/GVS147/) and SNPinfo (http://snpinfo.niehs.nih.gov/). DNA was extracted using AxyPrep Blood Genomic DNA Miniprep Kit (Axygen, Union City, CA, USA). Genotyping of each SNP was conducted using the MassARRAY platform (Sequenom, CA, USA) and performed by Bio Miao Biological Technology (Beijing) Co., Ltd.

### Statistical analysis

We tested each SNP for Hardy-Weinberg disequilibrium (HWD). Categorical variables were represented as frequency (percentage) and compared using the χ2 test. Continuous variables with normal distributions were represented as the mean ± standard deviation (SD) and compared using t-tests. Associations between SNPs and low response to HBV vaccine were analyzed via unconditional logistic regression with adjustment for possible confounders. The odds ratios (ORs) and 95% confidence intervals (CIs) were calculated to estimate risk. Ab titers in different groups were compared using analysis of covariance and adjusted for possible confounders. All statistical analyses were performed with the Statistical Package for Social Science (SPSS Inc., Chicago, IL, version 18.0). *P* < 0.05 was considered statistically significant.

## Results

### Characteristics of subjects

A total of 709 samples were included in the final analysis. Comparing the characteristics of low- and high responders, a higher percentage of breastfeeding (*P* = 0.013) and a higher level of Ab titers were observed in high responders (*P* < 0.001); data shown in Table 1.

**Table 1:** Characteristics of subjects in low- and high responders

Variants	Low responders (*N* = 41)	High responders (*N* = 668)	*P*
Gestational age (month)	39.0±1.4	38.9±1.4	0.761
Birth weight (kg)	3.4±0.4	3.4±0.5	0.533
Delivery mode (natural)	16(39.0%)	178(26.6%)	0.084
Gender (male)	21(51.2%)	353(52.8%)	0.840
Feeding pattern (breast)	8(19.5%)	259(38.8%)	0.013
Ab titers (mIU/ml)	1.6±0.4	3.2±0.5	< 0.001

Ab titers were transformed to their logarithms

### Genotype and allele distributions of SNPs

All of the SNPs in high responders were consistent with Hardy-Weinberg equilibrium (*P* > 0.05, Additional file 1). There were no significant differences in any of the eight SNP genotype or allele frequencies between low responders and high responders (Table 2). The *MAPK8* rs17780725 AA genotype seems to be associated with low response to hepatitis B vaccines, but it has not yet reached significance (*P* = 0.083).

**Table 2:** Genotype and allele distributions of SNPs between low- and high responders

Gene	SNPs	Genotype /allele	Low responders (N = 41)	High responders (N = 668)	*P*
TNF	rs1799964	CC	1(2.4)	33(4.9)	0.766
		CT	15(35.3)	235(35.2)	
		TT	25(61.0)	400(59.9)	
		C	17(20.7)	301(22.5)	0.705
		T	65(79.3)	1035(77.5)	
	rs1800629	AA	0(0.0)	1(0.1)	0.440^a^
		AG	5(12.2)	59(8.8)	
		GG	36(87.8)	608(91.0)	
		A	5(6.1)	61(4.6)	0.427^a^
		G	77(93.9)	1275(95.4)	
	rs3093671	AA	0(0.0)	1(0.1)	0.448^a^
		AG	5(12.2)	60(9.0)	
		GG	36(87.8)	606(90.9)	
		A	5(6.1)	62(4.6)	0.587^a^
		G	77(93.9)	1272(95.4)	
	rs769177	CC	36(87.8)	608(91.4)	0.424^a^
		CT	5(12.2)	56(8.4)	
		TT	0(0.0)	1(0.2)	
		C	77(93.9)	1272(95.6)	0.408^a^
		T	5(6.1)	58(4.4)	
	rs769178	GG	32(78.0)	519(78.4)	0.896
		GT	8(19.5)	133(20.1)	
		TT	1(2.4)	10(1.5)	
		G	72(87.8)	1171(80.9)	0.145
		T	10(12.2)	276(19.1)	
MAPK8	rs17780725	AA	5(12.2)	32(4.8)	0.083
		AG	12(29.3)	258(38.6)	
		GG	24(58.5)	378(56.6)	
		A	22(26.8)	322(24.1)	0.576
		G	60(73.2)	1014(75.9)	
	rs3827680	AA	8(19.5)	77(11.7)	0.213
		AG	15(36.6)	315(47.9)	
		GG	18(43.9)	266(40.4)	
		A	31(37.8)	469(35.6)	0.691
		G	51(62.2)	847(64.4)	

^a^Fisher exact text

### Association between SNPs and low response to hepatitis B vaccines

After adjustment for gestational age, birth weight, gender, delivery mode and feeding pattern, the rs17780725 AA genotype increased the risk of low response to hepatitis B vaccines (OR = 3.176, 95% CI: 1.137–8.869, Table 3). Additionally, subjects with the *MAPK8* rs17780725 AA genotype had a lower Ab titer than subjects with GA or GG carriers (*P* = 0.051, Table 4). Infants who were not breastfed had 2.901 times the odds of low response to the hepatitis B vaccine compared to infants who were breastfed. (95% CI:1.306–6.441).

**Table 3:** Associations between SNPs and low response to hepatitis B vaccines

Variables	OR	95%CI	*P*
Gestational age	1.046	0.777–1.408	0.767
Birth weight	1.000	0.999–1.001	0.541
Gender (male vs female)	1.080	0.568–2.054	0.815
Delivery mode (cesarean vs natural)	0.524	0.265–1.035	0.063
Feeding pattern (non-breast vs breast)	2.901	1.306–6.441	0.009
rs17780725 (AA vs GA + GG)	3.176	1.137–8.869	0.027

*P* values were calculated using multivariate logistic regression with the enter method, including SNP genotypes and all baseline covariates

**Table 4:** Ab titers in different genotypes of *MAPK8* rs17780725

SNP	Genotype	Ab titer	F	*P* ^a^
rs17780725	AA	2.95±0.72	3.825	0.051
	GA + GG	3.14±0.60		

Ab titers were log transformed. ^a^ *P* values were calculated with general linear regression with all baseline covariates

### Haplotype analysis

The pattern of pairwise linkage disequilibrium (LD) between the SNPs in *TNF* and *MAPK8* are shown in Additional file 2. The SNPs in *TNF* and *MAPK8* did not exhibit strong linkage disequilibrium, with no r^2^ greater than 0.8. Our haplotype analysis showed that no haplotype in *TNF* and *MAPK8* genes was significantly associated with low response to hepatitis B vaccines (Additional files 3 and 4).

## Discussion

Antibody responses in neonates are usually lower and differ from older children or adults [20, 21]. As a result, infants might have a lower response to HBV vaccination. Meanwhile, infants born to HBsAg(+) mothers have an increased risk of HBV infection compared to infants born to healthy mothers due to a persistent exposure to HBV from their mothers. It is therefore important to investigate the genetic risk factors for low response to hepatitis B vaccination and to improve the antibody level for these infants at high risk for HBV exposure.

The MAPK pathway is activated in innate immune cells in response to microbial infection [22]. MAPK8, also known as JNK1, may promote the phosphorylation of transcription factors following up-regulation in the expression of pro-inflammatory cytokines and chemokines. MAPK8 is mainly involved in the differentiation of helper T cells into Th1 cells during the antiviral immune response. As a result, different variants of the *MAPK8* gene could affect the Th1 mediated cellular immune response and ultimately affect the production of vaccine-induced antibodies [23]. Our study found that the *MAPK8* rs17780725 AA genotype may increase the risk of low response to hepatitis B vaccines. Rs17780725 is in strong LD with rs2698768 (r^2^ = 0.94), which is in the transcription factor binding site (TFBS) of *MAPK8*. This means that the replacement of G by A in rs17780725 might decrease the binding power between transcription factors and *MAPK8*, leading to down-regulation of *MAPK8* transcription and the MAPK8 protein, which could ultimately down-regulate the immune response to HBV vaccination. In fact, our results also show that subjects with the rs17780725 AA genotype had a lower level of Ab titers.

TNF is a pro-inflammatory cytokine that regulates innate immunity. When a vaccine-induced immune response occurs, TNF-α may increase the expression level of certain cytokines, leading to up-regulation of immune reaction [18]. Meanwhile, when HBV infection occurs, HBV-specific CD8^+^ T cells also secrete TNF-α in the body in order to clear HBV [24]. Dhiman N et al. has found the *TNF* rs1799964 CC genotype to be associated with a lower measles antibody response in a Somali population [25]. Berran Yucesoy et al. [18] also have shown the *TNF* rs1800629 AA genotype to be correlated with a lower antibody level after HBV vaccination in a non-Hispanic white childhood cohort. However, in our study, the SNPs in *TNF* were not found to be associated with low response to hepatitis B vaccination. Different ethnic groups may have different genetic backgrounds. For example, the MAF of rs1800629 in Han Chinese is quite different than that found in non-Hispanic whites (0.05 vs 0.11). Additionally, there were only 41 low responders in our study. The limited number of low responders may have limited statistical power. Thus, a similar study with larger sample size study should be conducted in the future.

Our study has some additional limitations. First, we only enrolled the infants of HBsAg(+)/HBeAg(−) mothers from our MTCT cohort. Infants born to HBsAg(+)/HBeAg(+) mothers were given injections of 20 μg hepatitis B vaccine at 0, 1 and 6 months of age in our cohort, and only 7 low responders were found among these infants. Although the dose of vaccine was the most important factor affecting the immune response to HBV vaccination, too few infants with a low response would limit statistical power. As a result, we only included the infants of HBsAg(+)/HBeAg(−) mothers in this study. Secondly, antibody levels decreased gradually during the follow-up period. For non-responders, a booster immunization still needs to be administered. Therefore, future studies should include a longer follow up time and an analysis of the association between SNPs and the administration of a booster dose.

## Conclusions

*MAPK8* polymorphisms are associated with immune responsiveness to HBV vaccinations in infants of HBsAg(+)/HBeAg(−) mothers.

## Additional files

Additional file 1: **Table S1.** Hardy-Weinberg equilibrium for SNPs in high responders. (DOCX 14 kb)

Additional file 2: **Figure S1.** Linkage disequilibrium of the SNPs in the TNF and MAPK8 genes. The color scale ranges from red to white according to r^2^ values (r^2^ values listed inside blocks). A, LD plot for *TNF*; B, LD plot for *MAPK8*. (DOCX 117 kb)

Additional file 3: **Table S2.** Associations between TNF gene haplotypes and risk of low response to hepatitis B vaccines. (DOCX 13 kb)

Additional file 4: **Table S3.** Associations between MAPK8 gene haplotypes and risk of low response to hepatitis B vaccines. (DOCX 13 kb)

## Abbreviations

Ab: Antibody
CIs: confidence intervals
CMIA: chemiluminescent microparticle immunoassay
GWAS: genome-wide association studies
HBeAg: hepatitis B e antigen
HBsAg: hepatitis B surface antigen
HBV: Hepatitis B virus
HLA: human leukocyte antigen
MAPK8: Mitogen-activated protein kinase eight
MTCT: Mother-to-child transmission
ORs: odds ratios
SD: standard deviation
SNP: single nucleotide polymorphism
TFBS: transcription factor binding site
TNF: Tumor necrosis factor
