# Supplementary material for: Mitogen-activated protein kinase eight polymorphisms are associated with immune responsiveness to HBV vaccinations in infants of HBsAg(+)/HBeAg(−) mothers
Source: BMC Infect Dis. 2018 Jun 14;18:274. doi: 10.1186/s12879-018-3166-x (PMC6000919; doi:10.1186/s12879-018-3166-x)
Supplement: Supplementary file 1 — Table S1. Hardy-Weinberg equilibrium for SNPs in high responders. (DOCX 14 kb) [file 12879_2018_3166_MOESM1_ESM.docx]

**Table S1. Hardy-Weinberg equilibrium for SNPs in high responders**

| **Rs number** | ***P*** |
| --- | --- |
| rs1799964 | 0.84 |
| rs1800629 | 0.73 |
| rs3093671 | 0.70 |
| rs769177 | 0.81 |
| rs769178 | 0.66 |
| rs17780725 | 0.15 |
| rs3827680 | 0.26 |
