# Supplementary material for: Mitogen-activated protein kinase eight polymorphisms are associated with immune responsiveness to HBV vaccinations in infants of HBsAg(+)/HBeAg(−) mothers
Source: BMC Infect Dis. 2018 Jun 14;18:274. doi: 10.1186/s12879-018-3166-x (PMC6000919; doi:10.1186/s12879-018-3166-x)
Supplement: Supplementary file 2 — Figure S1. Linkage disequilibrium of the SNPs in the TNF and MAPK8 genes. The color scale ranges from red to white according to r2 values (r2 values listed inside blocks). A, LD plot for TNF; B, LD plot for MAPK8. (DOCX 117 kb) [file 12879_2018_3166_MOESM2_ESM.docx]

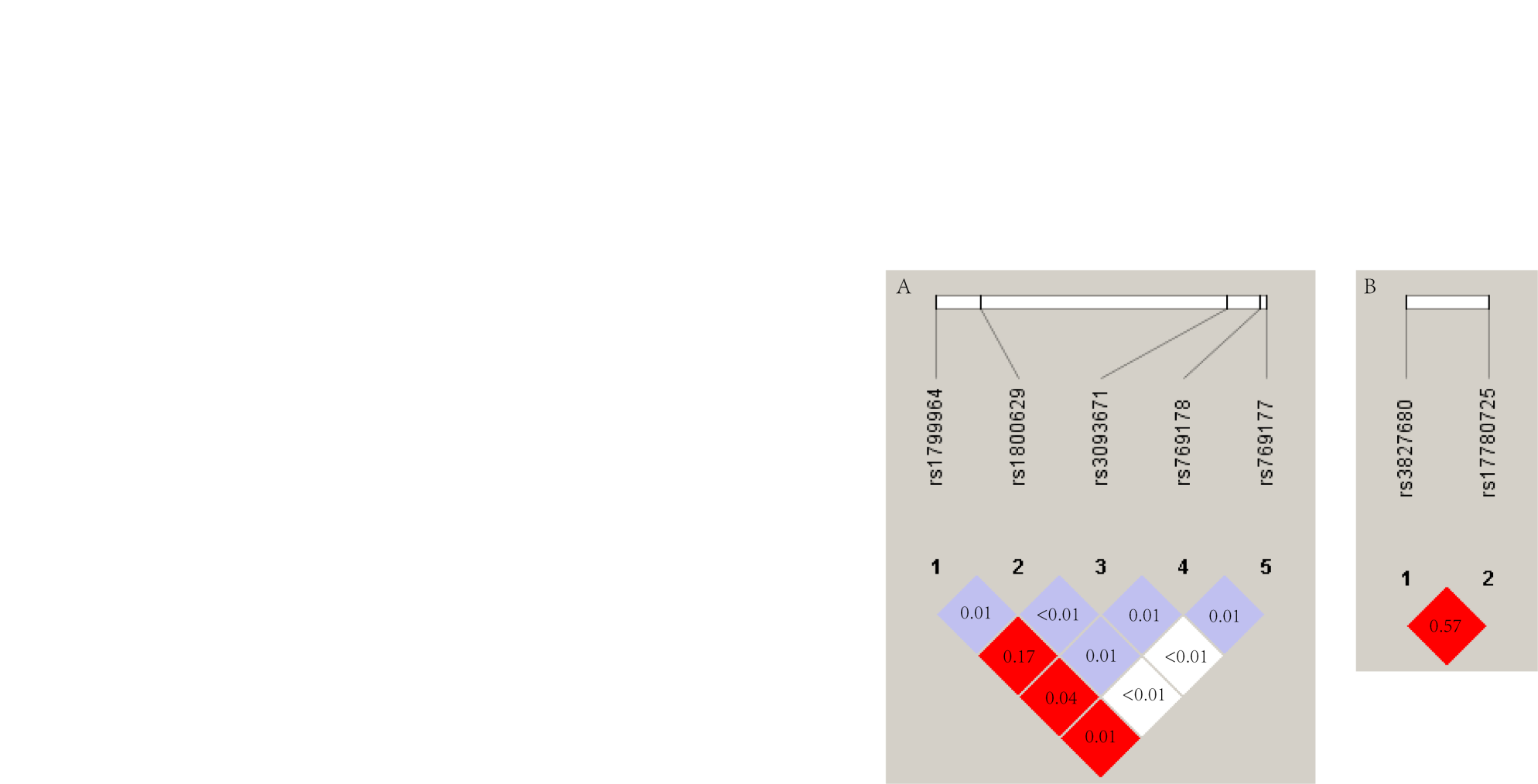


**Figure S1. Linkage disequilibrium of the SNPs in the TNF and MAPK8 genes.** The color scale ranges from red to white according to r^2^ values (r^2^ values listed inside blocks). A, LD plot for *TNF*; B, LD plot for *MAPK8*.
