# Supplementary material for: Mitogen-activated protein kinase eight polymorphisms are associated with immune responsiveness to HBV vaccinations in infants of HBsAg(+)/HBeAg(−) mothers
Source: BMC Infect Dis. 2018 Jun 14;18:274. doi: 10.1186/s12879-018-3166-x (PMC6000919; doi:10.1186/s12879-018-3166-x)
Supplement: Supplementary file 3 — Table S2. Associations between TNF gene haplotypes and risk of low response to hepatitis B vaccines. (DOCX 13 kb) [file 12879_2018_3166_MOESM3_ESM.docx]

**Table S2. Associations between TNF gene haplotypes and risk of low response to hepatitis B vaccines**

| **Haplotype** | **SNPs^a^** | | | | | **Frequency** | **OR (95% CI)** | ***P*** |
| --- | --- | --- | --- | --- | --- | --- | --- | --- |
|  | **1** | **2** | **3** | **4** | **5** |  |  |  |
| **1** | T | G | G | C | G | 0.5706 | 1.00 | - |
| **2** | C | G | G | C | G | 0.1753 | 0.83 (0.43 - 1.62) | 0.59 |
| **3** | T | G | G | C | T | 0.1158 | 1.10 (0.55 - 2.21) | 0.78 |
| **4** | C | G | A | C | G | 0.0472 | 1.39 (0.53 - 3.64) | 0.50 |
| **5** | T | A | G | C | G | 0.0448 | 1.47 (0.56 - 3.89) | 0.44 |
| **6** | T | G | G | T | G | 0.0446 | 1.43 (0.54 - 3.78) | 0.47 |
| **Rare^b^** | - | - | - | - | - | 0.0017 | - | - |

^a^ SNPs 1 to 5 were (in order) rs1799964, rs1800629, rs3093671, rs769177 and rs769178.

^b^ Rare: haplotypes with frequencies<0.01.
