# Supplementary material for: Mitogen-activated protein kinase eight polymorphisms are associated with immune responsiveness to HBV vaccinations in infants of HBsAg(+)/HBeAg(−) mothers
Source: BMC Infect Dis. 2018 Jun 14;18:274. doi: 10.1186/s12879-018-3166-x (PMC6000919; doi:10.1186/s12879-018-3166-x)
Supplement: Supplementary file 4 — Table S3. Associations between MAPK8 gene haplotypes and risk of low response to hepatitis B vaccines. (DOCX 13 kb) [file 12879_2018_3166_MOESM4_ESM.docx]

**Table S3. Associations between MAPK8 gene haplotypes and risk of low response to hepatitis B vaccines**

| **Haplotype** | **SNPs^a^** | | **Frequency** | **OR (95% CI)** | ***P*** |
| --- | --- | --- | --- | --- | --- |
|  | **1** | **2** |  |  |  |
| **1** | G | G | 0.6423 | 1.00 | --- |
| **2** | A | A | 0.2426 | 1.16 (0.68 - 1.96) | 0.58 |
| **3** | A | G | 0.1151 | 0.99 (0.48 - 2.04) | 0.97 |

^a^ SNPs 1 and 2 were rs3827680 and rs17780725 respectively.
